# Stress-Mediated Abnormalities in Regional Myocardial Wall Motion in Young Women with a History of Psychological Trauma

**DOI:** 10.3390/jcm12216702

**Published:** 2023-10-24

**Authors:** Assem Aweimer, Luisa Engemann, Sameh Amar, Aydan Ewers, Faegheh Afshari, Clara Maiß, Katharina Kern, Thomas Lücke, Andreas Mügge, Ibrahim El-Battrawy, Johannes W. Dietrich, Martin Brüne

**Affiliations:** 1 Bergmannsheil Bochum, Medical Clinic II, Department of Cardiology and Angiology, Ruhr University Bochum, 44789 Bochum, Germany; sameh.amar@bergmannsheil.de (S.A.); aydan.ewers@bergmannsheil.de (A.E.); faegheh.afshari@bergmannsheil.de (F.A.); andreas.muegge@rub.de (A.M.); ibrahim.elbattrawy2006@gmail.com (I.E.-B.); 2 LWL University Hospital Bochum, Division of Social Neuropsychiatry and Evolutionary Medicine, Ruhr University Bochum, 44791 Bochum, Germany; luisa-engemann@web.de (L.E.); clara.maiss@rub.de (C.M.); lisa.kern@rub.de (K.K.); martin.bruene@lwl.org (M.B.); 3 St. Josef-Hospital, University Hospital of Pediatrics and Adolescent Medicine, Department of Neuropediatrics and Social Pediatrics, Ruhr University Bochum, 44791 Bochum, Germany; luecke.thomas@rub.de; 4 Diabetes, Endocrinology and Metabolism Section, Department of Medicine I, Catholic Hospitals Bochum, St. Josef University Hospital Bochum, Ruhr University Bochum, 44791 Bochum, Germany; johannes.dietrich@rub.de; 5 Diabetes Centre Bochum/Hattingen, St. Elisabeth Hospital Blankenstein, Im Vogelsang 5–11, 45527 Hattingen, Germany; 6 Centre for Rare Endocrine Diseases, Ruhr Centre for Rare Diseases (CeSER), Ruhr University Bochum and Witten/Herdecke University, Alexandrinenstr. 5, 44791 Bochum, Germany; 7 Centre for Diabetes Technology, Catholic Hospitals Bochum, Gudrunstr. 56, 44791 Bochum, Germany

**Keywords:** risk factors, regional wall motion, speckle tracking echocardiography, stress cardiomyopathy, psychological trauma

## Abstract

Background: Psychosocial stress has been associated with the development and progression of atherosclerotic cardiovascular disease (CVD). Previously, we reported subtle differences in global longitudinal strain in somatically healthy women with a psychiatric diagnosis of borderline personality disorder (BPD). This study aimed to investigate the impact of BPD on segmental myocardial wall motion using speckle tracking echocardiography (STE) analysis. Methods: A total of 100 women aged between 18 and 38 years were included in this study. Fifty patients meeting the diagnostic criteria for BPD were recruited from the Department of Psychiatry (LWL-University Hospital Bochum) and compared with fifty age-matched healthy control subjects without previous cardiac disease. Laboratory tests and STE were performed with segmental wall motion analysis. Results: The BPD group had a higher prevalence of risk factors for CVD, with smoking and obesity being predominant, when compared with the control group. Other cardiovascular parameters such as blood pressure, glucose, and cholesterol levels were also elevated, even though not to pathological values. Moreover, in the STE analysis, the BPD group consistently exhibited decreased deformation in nine myocardial wall regions compared with the control group, along with a shift toward higher values in the distribution of peak pathological segments. Additionally, significantly higher values of free thyroxine concentration and thyroid’s secretory capacity were observed in the BPD group, despite falling within the (high-) normal range. Conclusions: BPD is associated with chronic stress, classical risk factors, and myocardial wall motion abnormalities. Further exploration is warranted to investigate the relationship between high-normal thyroid metabolism, these risk factors, and myocardial function in BPD patients. Long-term follow-up studies would be valuable in confirming the potential for predicting adverse events.

## 1. Introduction

Beyond traditional risk factors, psychosocial stress has been found to be associated with the development and progression of atherosclerotic cardiovascular disease (CVD) [1]. Not only does psychosocial stress have direct biological effects, but it is also strongly correlated with socioeconomic and behavioral risk factors for CVD [2]. Moreover, poor mental health is a risk factor for coronary heart disease even prior to the diagnosis of a mental disorder as well as during treatment, as it can arise both directly through biological pathways and indirectly through risky health behaviors [3]. Recently, the American Heart Association (AHA) highlighted the importance of placing greater emphasis on mental health in the context of CVD risk in a scientific statement [4]. This is supported by clear data showing that chronic stress, negative emotions such as anger and hostility, anxiety, depression, and pessimism can significantly increase the risk of myocardial infarction, stroke, or fatal CVD outcomes, with odds ratios ranging from 1.3 to 1.5 depending on the specific psychological risk factor [5,6,7,8,9]. Therefore, more integrative approaches that include psychological treatment for people at risk for CVD are needed.

In a recent study, we found small variations in global longitudinal strain (GLS) among physically healthy women diagnosed with borderline personality disorder (BPD) [10]. Furthermore, we found that approximately 17% of the individuals with BPD exhibited GLS values that were more than 3 standard deviations below the average, while only 4% of the participants in the control group showed such low values. The statistically significant but very slight difference suggests it is likely not clinically relevant, as such minor variations usually do not result in clinical abnormalities.

BPD is the most common personality disorder in the general population, with a prevalence rate of approximately 3% and a typical onset in adolescence or early adulthood [11,12]. BPD is characterized by intense, rapid mood swings, increased levels of impulsivity in at least two potentially self-harming domains, and difficulty maintaining stable interpersonal relationships. Recurrent emotional outbursts and inadequate levels of anger, both of which are difficult for patients to control, often lead to self-injurious and suicidal behavior [13]. Therefore, this severe mental illness is often associated with the aforementioned psychological risk factors for CVD and, given its prevalence in the general population, may serve as a model for studying the impact of mental illness on cardiovascular function.

Prior research has indicated that individuals diagnosed with BPD exhibit elevated prevalence rates of cardiovascular risk factors, such as hypertension, diabetes mellitus, and obesity [14,15]. Consistent with this risk profile, Moran et al. reported that patients with BPD have a 7.2-fold increased risk of coronary heart disease (CHD) and an 8.5-fold increased risk of stroke compared with the general population [16]. Recent work has further confirmed these findings, showing that the risk for cardiovascular disease is specific to BPD compared with other personality disorders, even when comorbid depression is taken into account [16,17].

While emotional stress has been shown to trigger characteristic patterns of segmental myocardial contractility decline in Takotsubo syndrome (TTS) [18], it remains unclear whether similar changes in segmental myocardial wall motion can be detected in patients with BPD. To address this question, we conducted a study analyzing segmental wall motion in BPD patients using echocardiographic speckle tracking analysis.

## 2. Materials and Methods

A detailed description of the study population can be found elsewhere [10]. In summary, we recruited a total of 100 women, aged 18 to 38 years, between March 2019 and October 2020. One group comprised 50 female inpatients from the Department of Psychiatry (LWL-University Hospital Bochum) who met the diagnostic criteria for BPD as defined by a structured clinical interview based on the Diagnostic and Statistical Manual of Mental Disorders, Fourth Edition (DSM-IV) criteria (German version 24). For comparison, 50 healthy female control subjects without previous cardiac disease were recruited. To ensure that the healthy control subjects had no history of psychiatric illness or current mental health problems, the MINI-DIPS 25 was administered as a brief diagnostic interview. Exclusion criteria for both groups were preexisting cardiovascular disease, male sex, and age greater than 40 years, so that the two groups were matched for sex and age. All participants gave written informed consent. The study was approved by the Ethics Committee of the Medical Faculty of the Ruhr University Bochum, Germany.

### 2.1. Echocardiography

In order to obtain images for two-dimensional speckle tracking echocardiography (STE), transthoracic echocardiography was performed on all participants following the recommendations of the EACVI/ASE/Industry Task Force on standardization of deformation imaging [19]. The examination was conducted by a skilled cardiologist (holding the highest qualification according to the German Society for Ultrasound in Medicine (DEGUM)) using a conventional two-dimensional gray-scale echocardiography system (GE Vivid E95; GE Healthcare, Solingen, Germany). Participants were positioned in the left lateral position. Image acquisitions necessary for subsequent speckle tracking analysis, including the calculation of global longitudinal strain, consisted of apical 2-, 3-, and 4-chamber views with continuous electrocardiographic recording. All images were obtained with standard settings for later STE analysis [20]. The entire ultrasound examination was digitally recorded, anonymized, and stored in the local database of BG University Hospital Bergmannsheil Bochum for offline analysis.

### 2.2. Speckle Tracking Analysis

For semiautomated two-dimensional speckle tracking analysis, each recording was processed with dedicated acoustic tracking software (GE EchoPAC version 203, GE Healthcare, Germany, Solingen, Germany).

All measurements were conducted in accordance with the guidelines for two-dimensional speckle tracking provided by the European Association of Cardiovascular Imaging (EACVI) and the American Society of Echocardiography (ASE)/Industry Task Force [21], under the supervision of two experienced cardiologists following the four-eyes principle.

Due to its representation of myocardial shortening in comparison with the baseline length, global longitudinal strain (GLS) of the left ventricle is reported as a negative value. When analyzing speckle tracking data, especially for categorizing GLS within a reference value range, only studies that established their standard values using the General Electric (GE) device manufacturer were considered. This approach was taken due to potential variations among providers and software. According to the American Society of Echocardiography (ASE) and the European Association of Cardiovascular Imaging (EACVI), a GLS value less negative than −20% is indicative of regional functional irregularities in the myocardium. Reference values for global and segmental longitudinal strain were obtained from the HUNT study [22].

### 2.3. Serum Markers and Calculated Biomarkers

A single serum tube and one EDTA tube of blood were obtained from each participant to undergo laboratory analysis. The following parameters were analyzed using a fully automated chemiluminescence-based system (DxI 800, Beckman-Coulter, Brea, CA, USA): Cholesterol, HDL cholesterol, LDL cholesterol, glycosylated hemoglobin (HbA1c), thyrotropin (TSH), free thyroxine (FT4), free triiodothyronine (FT3), and C-reactive protein (CRP). Intra-assay coefficients of variation (CV) for all parameters ranged between 2.0% and 5.0%, while inter-assay coefficients of variation (CV) ranged between 3.0% and 7.5%.

To evaluate the respective contributions of the pituitary and thyroid glands to variations in hormone concentrations, Jostel’s TSH index (JTI), thyroid’s secretory capacity (SPINA-GT), and the sum activity of peripheral deiodinases (SPINA-GD) were calculated based on steady-state concentrations of TSH, thyroid hormones, and constants for plasma protein binding and kinetics, respectively. These calculations were performed according to recent recommendations for thyroid trial design [23,24].

### 2.4. Data Analysis

All analyses were performed using IBM SPSS Statistics for Windows, version 26 (IBM Corp., Armonk, NY, USA) and R for macOS version 4.2.3 with the packages MASS, ellipse, and SPINA, and a significance level of *p* < 0.05 was selected for all tests. Data are presented as median (interquartile range (IQR)) for continuous variables with a non-normal distribution and as frequency (%) for categorical variables. Mean ± SEM is presented for continuous variables with a normal distribution. Normal distribution was assessed using the Kolmogorov test. Comparisons of categorical variables were conducted with the Pearson chi-square test, and continuous variables were analyzed with Student’s *t*-test and the Mann–Whitney U test.

## 3. Results

### 3.1. Baseline Characteristics

Table 1 shows the baseline characteristics of the BPD patients and healthy control subjects. Significant differences were found for all parameters except age, troponin concentration, and a subset of calculated biomarkers of thyroid homeostasis. No correlation was observed between the baseline characteristics, including laboratory parameters, and the STE analysis for specific wall regions. Three of the BPD patients suffered from hypothyroidism with L-thyroxine replacement therapy, as reported in Engemann et al., 2022 [10]. Because the groups were matched, there was no significant difference in age. Because of poor image quality, eight images from the BPD group and one image from the control group had to be excluded from the speckle tracking analysis. The high exclusion percentage in the BPD group resulted from the significantly increased BMI of the subjects.

### 3.2. Echocardiographic Results

Table 2 presents the echocardiographic measurements for both BPD patients and healthy controls. The BPD group showed a significantly thicker septum compared with the control group, although the thickness of 8.5 mm is still within the normal range. On the other hand, the tricuspid annular plane systolic excursion (TAPSE) values were higher in the control group, but these values were also within the normal range. Global longitudinal strain (GLS) was significantly lower in the control group, which was shown and discussed in the previous study [10].

**Figure 1:**
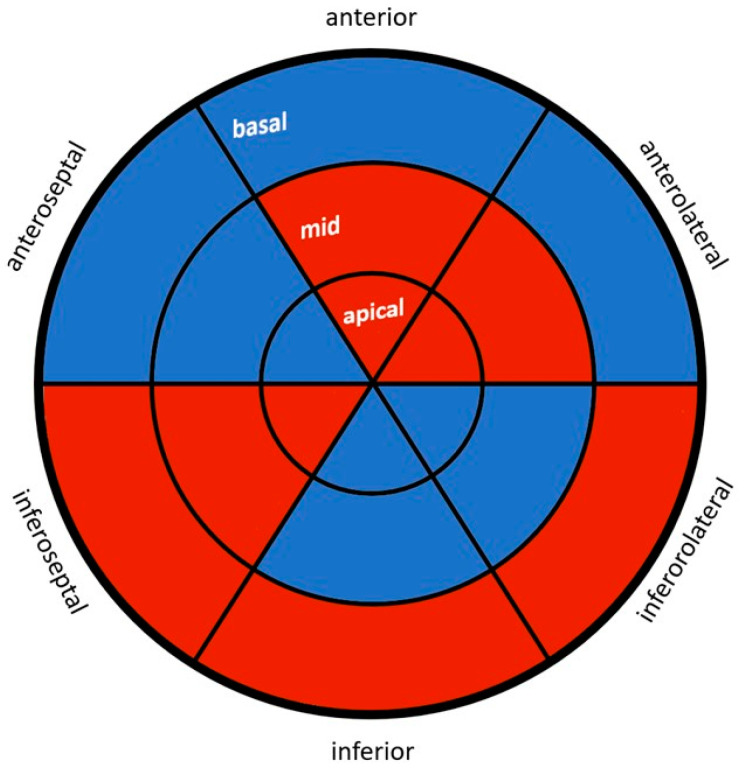
This figure shows all myocardial segments (bull’s eye). Segments highlighted in blue demonstrate a reduction in myocardial deformation within the BPD group when compared with the control group, whereas red-highlighted segments indicate that there is no reduction in deformation within the BPD group in comparison with the control group.

Overall, the BPD group had consistently lower values in the echocardiographic measurements and showed significant changes in nine regions compared with the control group. Figure 1 highlights the affected wall regions in the ‘’bull’s eye’’ format.

**Figure 2:**
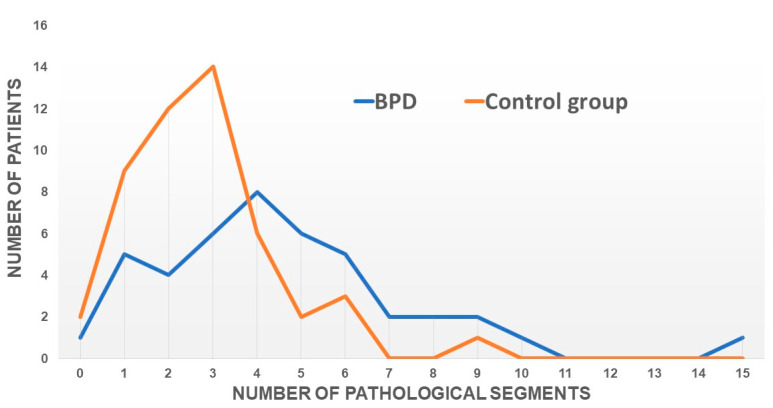
Frequency distribution of the number of myocardial segments with reduced wall motion in BPD patients compared with healthy controls. The BPD cohort shows a higher prevalence of pathological segments, as indicated by a rightward shift in the frequency distribution.

In addition, we compared each region of the 18 segments of the left ventricle for each subject with the normal values reported according to the HUNT study [22]. If any value was below the normal value, we considered this segment to be pathological. Subsequently, the number of pathological segments was counted per subject regardless of their location and this was graphically displayed (Figure 2). The peak of the number of pathological segments for the BPD group was shifted toward higher values compared with the control group.

## 4. Discussion

This is the first prospective study to assess wall motion abnormalities in patients with BPD. We detected a significant reduction in myocardial wall motion in patients with BPD compared with healthy controls. BPD is characterized by a history of childhood trauma and chronic stress in young female adults with a higher risk for coronary heart disease (CHD) [16,17]. In our BPD patient cohort, neither anamnestic nor clinical parameters suggest the manifestation of CHD. However, risk factors for CHD were significantly more present in the BPD patients, predominantly the high percentage of smokers and obesity. In previous research, it was observed that obese individuals with a very high BMI exhibited reduced global longitudinal strain (GLS) when assessed using speckle tracking echocardiography, suggesting subclinical cardiac dysfunction [25]. This observation was similarly noted in individuals with chronic cigarette smoking, although the effect on GLS was not as pronounced as the aforementioned obesity effect [26]. In our previous research on GLS, we also found a significant, albeit small, reduction in GLS in BPD patients [10]. The presence of well-established cardiovascular risk factors such as obesity and cigarette smoking being common in individuals with BPD may account for our observation of abnormal myocardial deformation. This suggests that BPD could act as a confounding factor, potentially explaining the observed changes, primarily driven by the higher prevalence of obesity and cigarette smoking in this patient cohort. Other cardiovascular parameters that may become risk factors, such as blood pressure, glucose, or cholesterol levels, were also elevated, even though not to pathological values.

Subtly reduced myocardial wall motion is often detected in CHD, in other specific cardiomyopathies, or during chemotherapy [27,28]. These observations are a result of direct myocardial damage due to reduced perfusion, tissue remodeling, or drug toxicity. Furthermore, in specific diseases, reduced myocardial wall motion detected with speckle tracking analysis can predict clinical outcomes before the manifestation of the disease [29,30]. However, speckle tracking in psychiatric patients for the prediction of cardiovascular diseases has barely been studied [10]. As stated in our previous study, BPD patients deserve a follow-up with speckle tracking analysis as a contribution to the primary prevention of CVD in susceptible individuals.

The hypothesis that psychological stress may contribute to altered myocardial wall motion warrants consideration. On the one hand, stress levels are measurably increased in BPD patients [10]. On the other hand, stress can directly impair cardiac contractility, as seen in Takotsubo syndrome (TTS) [18], which is characterized by a typical decrease in myocardial contractility in distinct wall regions of the myocardium. In our study, we did not observe a clear pattern of mainly affected regions, although the inferior wall segments appeared to be less affected. The distribution pattern does not offer a straightforward pathophysiological mechanism such as coronary blood flow. It might be the result of overlapping response patterns. Measurement-related misinterpretation is also possible and should ideally be addressed in future studies through examiner-independent assessments such as cardiac MRI. Interestingly, Rosman et al. reported on a cumulative impact of stressful life events on the development of TTS, suggesting that not only a single distinct event can trigger an acute decrease in myocardial contractility but also the quantity contributes to disease susceptibility, which can be assumed in BPD [31].

In a previous study, we identified a stress-induced type of TTS that mainly affected apical patterns of the myocardium and was characterized by an adapted thyroid metabolism via the hypothalamic–pituitary–thyroid axis [32]. Templin et al. (2015) demonstrated hypoconnectivity of the central brain regions associated with autonomic functions and regulation of the limbic system in patients with TTS [33]. We hypothesize a link between BPD and TTS due to reported alterations in the responsivity of the hypothalamic–pituitary–adrenal axis and an increase in inflammatory cytokines caused by early life stress, such as sexual or emotional abuse in childhood [34,35]. Additionally, we observed significantly higher values of FT4 concentration and thyroid’s secretory capacity (SPINA-GT) in the BPD group, even though they were still within the (high-) normal range. It is noteworthy that there was no observed correlation with myocardial wall deformation in the STE analysis. However, a comparative study observed a significant reduction in GLS in female patients with subclinical hyperthyroidism [36]. In one study, it was found that aggressive behavior in BPD patients was correlated with FT3 levels. However, no cardiac parameters were assessed in these patients [37]. Surprisingly, in BPD, SPINA-GT was elevated, rather than markers of the central set point of the feedback loop as expected. This observation may reflect dynamical compensation, i.e., increased thyroid volume in the setting of chronic stimulation due to lifelong stress [38,39]. Published data suggest an association between thyroid hormone concentrations and the risk of sudden cardiac death (SCD) even in euthyroid populations with high-normal FT4 concentration [40,41]. If confirmed in future studies, this observation may help clarify the well-known mortality gap (with higher rates of SCD) between psychiatric patients and the general population, which is caused by several factors such as psychotropic drugs or cardiovascular risk factors.

Whether high-normal thyroid metabolism can be attributed to these risk factors needs to be clarified in further studies. Such a relationship is suggested, however, by a positive association between physiological dysregulation and certain biomarkers of thyroid homeostasis [42]. Regarding the prediction of arrhythmic events using strain echocardiography, previous studies have examined changes in the mechanical dispersions of the left ventricle [43,44]. Our data, based on strain results, do not suggest an increased risk of arrhythmia in patients with BPD, especially when considering the mechanical dispersion of the LV. This finding aligns with the electrocardiographic results we obtained, including repolarization parameters, which typically indicate a higher likelihood of ventricular arrhythmia [45].

Psychiatrists often collaborate with cardiologists regarding QTc-prolonging drugs and ECG abnormalities. Our findings suggest that monitoring cardiac function in psychiatric patients exposed to psychological stress could serve as an additional tool for identifying patients at risk, although further long-term investigations are necessary to assess the precise significance of a latent myocardial wall motion abnormality detected through speckle tracking echocardiography.

The significant reduction in BNP levels in the predominantly obese BPD group is most likely derived from the known paradox of low BNP levels in obesity. This phenomenon has not been fully elucidated yet [46].

The present study has several limitations. Echocardiography is an operator-dependent diagnostic tool and may be challenging at times, even in experienced hands. Pathological values have been observed even in supposedly mentally healthy subjects, such as the control group. However, all patients in our study were young women, mainly with slim figures in the control group, which may have contributed to the feasibility of echocardiography due to their constitution. We had to exclude eight patients from the BPD group and one patient from the control group due to inadequate echocardiogram image quality. Nonetheless, poor image quality is a prevalent issue in echocardiography, crucial for correct and reproducible speckle tracking analysis [47]. Furthermore, our study exclusively enrolled female participants, thus limiting the generalizability of our findings to both biological sexes. The primary rationale for this exclusion was the frequent co-occurrence of other personality disorders, such as narcissistic and antisocial personality disorders, in male patients with BPD. In the context of multiple factors potentially impacting myocardial wall motion, it could be beneficial to conduct a multivariable analysis incorporating BPD and potential confounders. However, due to a small sample size of 50 in each group, this could not be feasibly implemented in this study. An alternative approach could be to match subjects in both groups for major predictors, which would necessitate an even larger sample size, particularly during the screening phase. Carefully planned future studies could address this question as long as they include significantly larger cohorts.

## 5. Conclusions

In summary, individuals with BPD often experience persistent stress and exhibit classical risk factors associated with myocardial wall motion abnormalities. Further research is needed to determine the extent to which these findings can provide prognostic insights. Conducting long-term follow-up studies could be of interest and may help validate the potential for predicting adverse events.

Clinical implication section: Speckle tracking echocardiography (STE) may have the potential for identifying latent myocardial wall motion irregularities in individuals with BPD, especially those under mental stress. Analyzing cardiac function using STE in psychiatric patients experiencing elevated mental stress levels could complement traditional parameters and aid in the identification of at-risk patients.

## Figures and Tables

**Table 1:** Baseline characteristics of included persons.

	BPD (*n* = 50)	Control Group (*n* = 50)	*p* Value
Age	23.85 (±5.2)	24.10 (±3.9)	n.s.
Pack years	3.9 (±6.0)	0.2 (±1.1)	<0.001 *
SBP (mmHg)	126.9 (±12.8)	115.5 (±9.5)	<0.001 *
DBP (mmHg)	82.9 (±9.5)	75.8 (±7.3)	<0.001 *
WtH ratio	0.77 (±0.1)	0.74 (±0.04)	<0.001 *
BMI (kg/m^2^)	28.37 (±6.6)	22.40 (±3.0)	<0.001 *
Height (cm)	167.4(±6.3)	166.7(±5.6)	0.53
Cholesterol/HDL ratio	3.29 (±1.2)	2.77 (±0.55)	0.004 *
HDL (mg/dL)	55.94 (±15.6)	71.68 (±16.4)	<0.001 *
HbA1c (%)	5.2 (±0.29)	4.9 (±0.22)	<0.001 *
CRP (mg/dL)	0.47 (±0.79)	0.17 (±0.33)	0.010 *
TSH (mIU/L)	1.45 (±0.68)	1.82 (±0.78)	0.012 *
fT3 (pmol/L)	5.41 (±0.69)	5.11 (±0.45)	0.012 *
fT4 (ng/L)	8.46 (±1.30)	7.54 (±1.16)	<0.001 *
SPINA-GT (pmol/s)	2.82 (±0.17)	2.17 (±0.14)	<0.001 *
SPINA-GD (nmol/s)	46.89 (±1.14)	49.39 (±1.17)	n.s.
JTI	1.73 (±0.07)	1.79 (±0.07	n.s.
BNP (pg/mL)	17.41 (±9.59)	29.02 (±18.97)	<0.001 *
hsTroponine (pg/mL)	2.42 (±0.46)	2.80 (±2.71)	0.334
PR (ms)	139 (±22.7)	141.7 (±25.1)	0.62
QRS (ms)	83.6 (±13.3)	87 (±11.8)	0.18
QTc (ms)	403.7 (±31.2)	402.3 (±25.7)	0.80
Tpe (ms)	62.3 (±11.4)	61.4 (±13.3)	0.74

Data are presented as mean (±SEM), unless otherwise indicated. SBP, systolic blood pressure; DBP, diastolic blood pressure; WtH ratio, waist-to-hip ratio; BMI, body mass index; HDL, high-density lipoprotein; CRP, C-reactive protein; TSH, thyroxine-stimulating hormone; fT3, free triiodothyronine; fT4, free thyroxine; JTI, Jostel’s TSH index; SPINA-GT, thyroid’s secretory capacity; SPINA-GD, sum activity of peripheral deiodinases; BNP, brain natriuretic peptide; hsTroponin, high-sensitivity troponin; PR, electrocardiographic period from beginning of P wave to onset of QRS complex; QRS, electrocardiographic period from beginning of Q wave to end of S wave; QTc, electrocardiographic period from beginning of Q wave to end of T wave corrected to heart rate using Bazett’s formula; Tpe, electrocardiographic period from peak to end of T wave. * *p* < 0.05.

**Table 2:** Echocardiographic parameters with segmental results of longitudinal strain.

	BPD (*n* = 42)	Control Group (*n* = 49)	*p*-Value
PW (mm)	8.0 (±1.3)	7.7 (±1.2)	0.240
IVS (mm)	8.5 (±1.1)	7.5 (±1.5)	<0.001 *
Heart rate (bpm)	72 (±12)	69 (±13)	0.259
LVEDD (mm)	45.9 (±3.8)	45.9 (±4.7)	0.990
LVESD (mm)	29.6 (±3.6)	28.0 (±5.1)	0.076
LVEDV (mL)	90.6 (±24.6)	91.6 (±17.4)	0.806
LVESV (mL)	37.7 (±10.2)	38.2 (±8.3)	0.788
SV (mL)	52.5 (±14.4)	53.3 (±11.2)	0.739
LVEF (%)	58.1 (±4.1)	58.3 (±4.5)	0.860
TAPSE (mm)	22.8 (±4.3)	24.7 (±3.7)	0.019 *
LV mass (g)	117.3 (±42)	109.3 (±41)	0.02
LV mass-index (g/m^2^)	61.9 (±21)	65.5 (±22)	0.81
RWT	0.35 (±0.06)	0.56 (±1.6)	0.32
GLS (%)	−19.04 (±1.9)	−20.7 (±1.7)	<0.001 *
LV Mechanical dispersion (ms)	35.2 (±8.5)	39.5 (±13.9)	0.30
Anteroseptal			
Basal (%)	−16.5 (±2.8)	−18.1 (±3.2)	0.01 *
Mid (%)	−19.5 (±2.6)	−21.8 (±3.0)	<0.001 *
Apical (%)	−23.2 (±4.2)	−27.0 (±4.8)	<0.001 *
Inferolateral			
Basal (%)	−18.8 (±3.8)	−19.6 (±4.5)	0.369
Mid (%)	−18.0 (±3.2)	−20.7 (±3.3)	<0.001 *
Apical (%)	−18.9 (±4.3)	−21.5 (±4.9)	0.01 *
Inferoseptal			
Basal (%)	−19.6 (±3.7)	−20.9 (±4.1)	0.126
Mid (%)	−18.8 (±8.2)	−20.4 (±3.1)	0.191
Apical (%)	−23.3 (±4.3)	−24.6 (±4.6)	0.171
Anterolateral			
Basal (%)	−17.7 (±5.0)	−19.8 (±4.7)	0.048 *
Mid (%)	−19.3 (±4.1)	−20.3 (±4.8)	0.333
Apical (%)	−18.7 (±5.0)	−18.9 (±5.7)	0.842
Inferior			
Basal (%)	−17.9 (±4.4)	−19.9 (±4.1)	0.028
Mid (%)	−18.4 (±2.9)	−20.6 (±3.3)	0.001 *
Apical (%)	−20.4 (±3.4)	−23.3 (±4.9)	0.001 *
Anterior			
Basal (%)	−16.0 (±3.1)	−17.9 (±3.5)	0.007 *
Mid (%)	−21.1 (±4.1)	−21.9 (±3.3)	0.315
Apical (%)	−21.4 (±4.3)	−23.0 (±5.4)	0.137

Data are presented as mean (±SEM), unless otherwise indicated. PW, posterior wall in parasternal long axis; IVS, interventricular septum in parasternal long axis; LVEDD, left ventricular end-diastolic diameter; LVESD, left ventricular end-systolic diameter; LVEDV, left ventricular end-diastolic volume; LVESV, left ventricular end-systolic volume; SV, stroke volume; LVEF, left ventricular ejection fraction; TAPSE, tricuspid annular plane systolic excursion; RWT, relative wall thickness of the left ventricle; GLS, global longitudinal strain. * *p* < 0.05.

## Data Availability

The datasets used for the analysis in the current study are available from the corresponding author on reasonable request.
